# Rules for the Sick Room

**Published:** 1856-09

**Authors:** 


### RULES FOR THE SICK ROOM.

NEVER place yourself between the patient and the fire, for there is always a current in that direction from all parts of the room, hence the effluvia from the sick man passes by, and is breathed by you.

Never swallow the saliva, nor eat or drink anything in a sick room.

Do not go where the sick are while in a perspiration nor under any circumstances of exhaustion.

In your visits to the sick, in pity, be brief.

In watching with sick people, eat a regular meal before you go into the room, and repeat at intervals of not over four hours, this keeps the stomach in a state of excitement, which repels infection.

Speak kindly, cheerfully, encouragingly to the sick

In waiting upon them study the happy mean in anticipating their wants, without being annoyingly officious.

Do not stare at a sick man, nor show a surprised countenance, and speak softly, with distinctness.

